# Hematopoietic Stem Cells: Nature and Niche Nurture

**DOI:** 10.3390/bioengineering8050067

**Published:** 2021-05-15

**Authors:** Geoffrey Brown

**Affiliations:** Institute of Clinical Sciences, School of Biomedical Sciences, College of Medical and Dental Sciences, University of Birmingham, Edgbaston, Birmingham B15 2TT, UK; g.brown@bham.ac.uk; Tel.: +44-0-121-414-4082

**Keywords:** hematopoietic stem cells, decision-making, stem cell niches, cytokines

## Abstract

Like all cells, hematopoietic stem cells (HSCs) and their offspring, the hematopoietic progenitor cells (HPCs), are highly sociable. Their capacity to interact with bone marrow niche cells and respond to environmental cytokines orchestrates the generation of the different types of blood and immune cells. The starting point for engineering hematopoiesis ex vivo is the nature of HSCs, and a longstanding premise is that they are a homogeneous population of cells. However, recent findings have shown that adult bone marrow HSCs are really a mixture of cells, with many having lineage affiliations. A second key consideration is: Do HSCs “choose” a lineage in a random and cell-intrinsic manner, or are they instructed by cytokines? Since their discovery, the hematopoietic cytokines have been viewed as survival and proliferation factors for lineage committed HPCs. Some are now known to also instruct cell lineage choice. These fundamental changes to our understanding of hematopoiesis are important for placing niche support in the right context and for fabricating an ex vivo environment to support HSC development.

## 1. Introduction

HSCs produce a wide range of cell types, each with unique functions, that include platelets, erythrocytes, basophils/mast cells, eosinophils, neutrophils, dendritic cells, B lymphocytes, innate lymphoid cells, and T lymphocytes. For some of these major cell lineages, for example, dendritic cells, B lymphocytes, innate lymphoid cells, and T lymphocytes, there are sub-populations of cells with a more precise function. The main cell types, with the exception of the mature T lymphocytes, are generated in the adult by the bone marrow. An engineered marrow-like environment for HSCs should enable the extensive multipotent nature of HSCs. A further requirement is the need to simulate both the steady state production of the blood cell types and the increased production of a particular type of cell during emergency hematopoiesis. For example, there is an urgent need to generate, megakaryocytes giving rise to platelets in the case of thrombocytopenia, and neutrophils to control a bacterial infection.

The responsiveness of cells to their environment is crucial to the cohesion of multicellular organisms and to meeting an organism’s changing demands. For the many types of cells, the environmental controls on cell survival versus cell death ensure proper cell numbers and also eliminate cells that are in the wrong place [1]. Similarly, building an ex vivo model of hematopoiesis is contingent on an understanding of the ways HSCs and HPCs interact with their neighboring cells [2] and respond to the hematopoietic cytokines [3]. Neighboring cells are heterogeneous and there are more than 50 cytokines that regulate hematopoietic cell differentiation in some way. Needless to say, the complexity of the controls on HSCs, their development and the survival and expansion of developing cells, during steady-state versus emergency hematopoiesis, places substantial demands on engineering hematopoiesis ex vivo. The current state of the research field is that faithfully replicating steady-state and emergency hematopoiesis is not yet possible. Therefore, this review examines some of the key considerations and constraints.

## 2. HSCs Are a Heterogeneous Population of Cells

**Figure 1:**
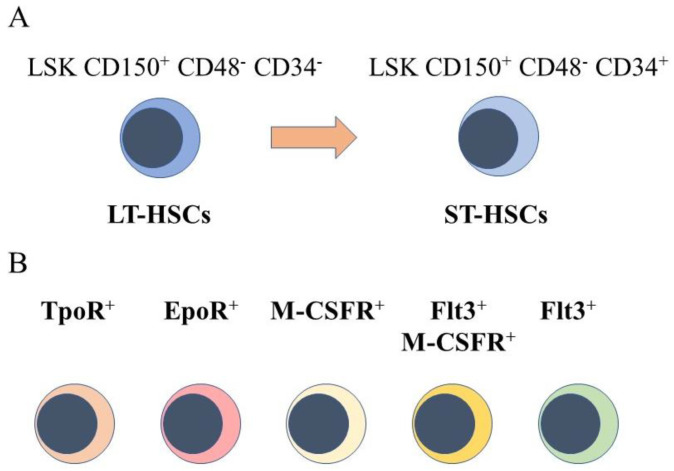
Murine hematopoietic stem cell populations. (**A**) mouse hematopoietic stem cells (HSCs) can be divided into cells that reconstitute severe combined immunodeficient mice long term (LT-HSCs) and short term (ST-HSC) and identified by using panels of cell surface markers. (**B**) sub-populations express the receptor for thrombopoietin at their cell surface(TpoR^+^), mRNA for the receptor for erythropoietin (Epo), the cell surface receptor for macrophage colony-stimulating factor (M-CSFR^+^), this receptor together with the cell surface receptor fms-like tyrosine kinase 3, and the fms-like tyrosine kinase 3.

The starting point for engineering hematopoiesis ex vivo must be knowledge about the nature of HSCs. In a strict sense, HSCs are able to reconstitute the entire blood and immune system when transplanted into a severe combined immunodeficient (SCID) mouse [4] and newer mouse models [5]. HSCs that are termed long term reconstituting HSCs (LT-HSC) have an extensive self-renewal capacity and differentiate into HSCs that reconstitute hematopoiesis in the short term, namely short term reconstituting HSCs (ST-HSC). A comprehensive view of mouse hematopoiesis, including the nature of stem and progenitor cell populations, is still well in advance of that established for human hematopoiesis because of the lack of cell surface markers. Various primitive mouse cell populations, including LT-HSC and ST-HSC, can be ring fenced and FACS sorted by means of cell surface markers. A small fraction of primitive bone marrow cells lacking cell lineage markers and that express Sca-1 and c-kit are termed Lineage^−^Sca1^+^c-Kit^+^ (LSK) cells. Within this population, for example, LT-HSCs are LSK CD150^+^ CD48^-^ CD34^-^ and ST-HSCs are LSK CD150^+^ CD48^−^ CD34^+^, whereby a gain of CD34 expression separates these populations (Figure 1A). A single CD34^-/low^ HSC is able to reconstitute hematopoiesis in mice [6]. Accordingly, a longstanding view is that HSCs are both homogeneous and multipotent. This reservoir of cells is largely quiescent in the G0 of cell cycle, self-renews, and HSCs are ‘selected’ to undergo differentiation.

However, sub-populations of murine HSCs have been identified that selectively express receptors for some of the cytokines that are lineage-affiliated. As tested by transplantation experiments in mice, each sub-set also mainly gives rise to cells of one cell type. A sub-population of HSCs that requires thrombopoietin for maintenance, and that expresses the megakaryocyte-affiliated von Willebrand factor, gave rise to a long-term platelet- or platelet/myeloid-biased reconstitution when transplanted as single cells [7]. The identification of repopulation-competent HSCs that express the megakaryocyte-restricted surface marker CD41 (alph11b integrin, platelet GP11b) further supported the existence of megakaryocyte-biased HSCs [8], and other workers identified such cells with impaired self-renewal [9]. Macrophage colony-stimulating factor (M-CSF) supports the survival and proliferation of macrophage progenitor cells. Around 20% of LT-HSC and ST-HSC were observed to express the receptor for macrophage colony-stimulating factor (M-CSFR) at their surface, and 1% and 3% of these cells, respectively, expressed this receptor together with the fms-like tyrosine kinase 3 (Flt3), for Flt3 ligand. The expression of Flt3 distinguishes lymphoid and myeloid progenitors from those of megakaryocytes and erythrocytes. Erythropoietin (Epo) supports the development of erythroid progenitors and mRNA for the Epo receptor was observed to be expressed by 13% of LT-HSCs and 20% of ST-HSC, and was rarely co-expressed with Flt3 mRNA [10]. Granulocyte colony-stimulating factor (G-CSF) and granulocyte/macrophage colony-stimulating factor (GM-CSF) are important for the development of granulocyte progenitor cells. G-CSF regulates the activity of transplantable HSCs, via activation of toll-like receptor signaling, and increases the frequency of HSCs in bone marrow [11]. The receptor for GM-CSF is expressed at low to moderate levels by primitive mouse HSCs [12].

Murine myeloid- and lymphoid-biased HSCs selectively express the cell surface markers CD41 and CD86, respectively. Myeloid-biased HSCs also express CD150 at a higher level and exclude the DNA-binding dye Hoechst 33342 more effectively [13,14] and as mice age become predominant [15]. The use of single-cell functional assays to examine the primitive CD34^+^ compartment of human adult bone marrow revealed mostly unipotent progenitors, with few oligo-potent progenitors. A shift occurs during the transition from in utero to adult hematopoiesis, because fetal liver contained large numbers of oligo-potent progenitors [16]. For human cord CD34^+^ blood cells, HSCs were FACS sorted as CD49f^+^ cells and their engraftment as single cells in NOD-scid-IL2Rgc^−/−^ (NSG) mice was optimized by intra-femoral injection. The frequency of multi-lineage chimerism was rather low for both first (28%) and second transfers (14%), in keeping with the heterogeneous nature of HSCs [17].

## 3. Bone Marrow Niche Cells Are Heterogeneous

Where HSCs reside in the bone marrow is crucial to how they change their behavior. Knowledge of the various non-hematopoietic marrow cells has advanced in recent years, but the precise nature of the cells that HSCs interact with and how they sustain or evoke a particular aspect of HSC behavior are not entirely clear. Whilst stromal cell cultures are able support the expansion of HSCs/HPCs [18], the roles(s) of sub-populations remains unclear, largely because the heterogeneity of stromal cells is unresolved due to a paucity of surface markers. However, and as has been known for decades, the bone marrow vasculature is key to the control of HSCs [2]. The endosteal region of bone marrow is highly perfused by arterioles and arterial capillaries. Quiescent HSCs reside around arterioles where perivascular, endothelial, Schwann, and sympathetic neuronal cells ensure the factors that are required to maintain HSCs and/or HPCs. For example, Schwann cells have been reported to promote HSC quiescence by activating transforming growth factor-β1 [19]. Sinusoids are dispersed uniformly within the marrow space. Activated HSCs reside close to sinusoidal niches that are likely to influence the behavior of HSCs. Stem cell factor, which is important to the survival and proliferation of HSCs, is largely expressed by the perivascular cells around sinusoids. An interesting proposition is that endosteal niches that are just outside of thick-walled arterioles provide a hypoxia environment that is conducive to HSC quiescence, whereas a vascular niche favors HSC proliferation and differentiation [20]. However, there is still a need to confirm the existence of a hypoxic niche.

Other niche cells for HSCs and HPCs include osteoblasts, chondroblasts/chondrocytes, and adipoblasts. These cells, and stromal cells, are thought to arise from mesenchymal stem and progenitor cells (MSCPs), but their lineage relationships to MSCPs remains unclear. Osteoblasts create bone, and chondroblasts and chondrocytes create and maintain cartilage, respectively. The mature adipocytes in white adipose tissue contain a lipid droplet for triglyceride storage. It is likely that the interaction of HSCs with particular MSCP-derived cells regulates HSC self-renewal and differentiation. A manipulated increase in the number of osteoblasts in bone marrow led to high levels of the Notch ligand jagged 1 and an increase in the number of HSCs [21]. Even so, jagged 1-dependent Notch signaling was shown to be dispensable for both the self-renewal and differentiation of HSCs [22]. Osteoblasts have also been proposed to support the function of HSCs via N-cadherin-mediated adhesion [23], but other workers have reported from knockout studies that osteoblastic N-cadherin is not important for HSC support [24]. Activated osteoblasts have been proposed to control the HSC pool size by producing osteopontin to control quiescence [25]. Osteoclast degradation of endosteal components promotes the localization of HSCs to endosteal regions [26]. The role of adipocytes in tissue regeneration, including wound healing, hair follicle regeneration, and mammary gland development, and within stem cell niches is an emerging theme [27]. Bone morphogenic protein and Tie2 signaling regulate HSCs and/or their offspring, with obesity in mice leading to an abundance of immune cell sub-sets, for example macrophages and lymphocytes.

As yet, prescribing the precise nature of the cellular niches that control the different aspects of HSC behavior and their development is difficult for various reasons. There is the need to develop markers to resolve the heterogeneity of some of the niche cell populations. As mentioned above, there are contradictions regarding whether particular cells and molecules do, or do not, have a key role. Moreover, we now know that HSCs are a heterogenous population of cells, and that this includes HSCs that are biased towards or affiliated to a lineage. There is therefore the need to re-write the nature of the various niches for HSCs.

## 4. Mapping HSC Developmental Progression

The complex cross-talk between HSCs and their neighboring cells is clearly important for how HSCs change their status to progress developmentally, and an understanding of HSC progression is crucial for placing niche support in the right context. For very many years, tree-like diagrams have been used to describe how HSCs give rise to each of the blood and immune end cell types. That HSCs first make a choice whether to develop along the pathways for either lymphoid or myeloid (all other cells) cells is a longstanding dichotomy model that is still favored. This dichotomy led to the concept of a common lymphoid progenitor and a common myeloid progenitor. Further progression is then by means of a prescribed number of preferred branching and binary steps towards the unipotent HPCs for each of the mature cell types [28].

**Figure 2:**
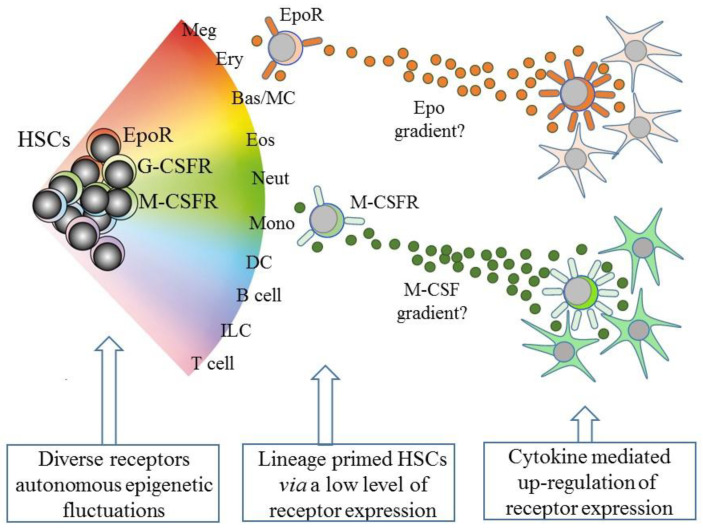
A continuum model of hematopoiesis and the instructive role of cytokines. Hematopoietic stem cells (HSCs) “choose” a lineage from a continuum of the variety of options and are a mixture of cells with different lineage signatures, shown by the different colors. HSC sub-populations express mRNA for the receptor for erythropoietin (EpoR) and the receptors for macrophage colony-stimulating factor (M-CSFR) and granulocyte colony-stimulating factor (G-CSFR) at low levels. Their cytokines can instruct erythroid, monocyte, and neutrophil fates, respectively, and may also provide a chemotactic gradient to attract cells to a niche. Macrophage-colony stimulating factor (M-CSF) and granulocyte-colony stimulating factor (see text) positively auto-regulate receptor expression. Meg, megakaryocyte; Ery, erythrocyte; Bas/MC, basophil/mast cell; Eos, eosinophil; Neut, neutrophil; Mon, monocyte; DC, dendritic cell; ILC, innate lymphoid cell.

However, and as above, the existence of lineage biased HSCs means that HSCs can affiliate directly to just a single pathway. Therefore, some models abandoned a tree-like depiction in favor of HSCs “choosing” a cell lineage from a continuous spectrum of all of the lineage fates. Such models, therefore, do not dictate stepwise routes to each end cell type, but there are still near-neighbor relationships between the cell lineages that are inferred from shared characteristics. From these premises, an early continuum model was the pairwise model (Figure 2) [29].

The developmental progression of HSCs is much more dynamic than a simple linear pathway towards an end cell type. There is flexibility to ensure that hematopoiesis is adaptable to the demand for a particular cell type(s). HSCs and HPCs that have “chosen” a cell lineage remain versatile as they can develop into a mature cell that does not belong to the initial choice. RNA sequencing of single mouse HSCs/HPCs revealed alternative trajectories [30]. Megakaryocyte-biased HPCs can step-sideways to erythropoiesis [31]. HPCs that are some way along a development pathway can still “change their mind” to adopt a different fate. The earliest T cell progenitors can still give rise to macrophages and natural killer cells [32]. There is also even interconversion of some end cell types. The various types of mature CD4^+^ T-cell effector cells produce different cytokines relating to their functionality. The sub-sets include T helper 1 cells, T helper 2 cells (Th2), interleukin 17-producing T helper cells (Th17), follicular T helper cells (Tfh), and regulatory T cells (iTreg). Th2 cells can convert to Tfh [33], iTreg to pro-inflammatory Th17 [34], and memory Th2 cells to iTreg [35], and cell state conversions are driven by the cytokine signals received [36].

## 5. Intrinsic Events Influence HSCs Lineage “Choice”

The existence of lineage-affiliated HSCs means that a distinction between HSCs and HPCs becomes somewhat redundant. Additionally, self-renewal, which has long been viewed as a cardinal property of HSCs, does not go strictly hand in hand with pluripotency. The repopulation competence of single lineage-affiliated HSCs indicates that they are likely to self-renew. In this scenario, fundamental questions about the fate of HSCs are: How do they “choose” a cell lineage, and do niches play a key role? Investigators have debated at length whether HSCs “choose” a cell lineage in a random and cell-intrinsic manner or whether they are instructed by cytokines, presumably presented by niche cells. Choosing either nature or nurture, respectively, is reductionist and overly simplistic. Longstanding and recent findings support the view that both of these influences drive HSCs to adopt a lineage fate.

Studies of the multipotent murine hematopoietic cell line, EML, revealed that transcriptome-wide noise plays a role in controlling lineage choice. Cells that expressed the stem cell marker Sca1 at either an extremely high or low level had distinct transcriptomes, and their unique gene expression profiles fluctuated, eventually reverting to that of the median cells. The profile lasted long enough to confer a propensity towards development along either the erythroid or myeloid pathways. The conclusion was that the protein level fluctuations that render this proclivity are a manifestation of “gene expression noise” [37]. Similarly, a Monte Carlo model of decision-making attributed the probability of HSCs adopting a lineage to noise within the dynamics of the expression of mRNAs, including decay, and promoter activities. These workers compared gene expression by individual self-renewing and erythroid-committed progenitors obtained from the cell line EML. They identified putative erythroid and myeloid commitment-associated genes, for example, for the transcription factors Gata1 and Gata2 and the receptor for Epo for erythropoiesis, and myeloperoxidase for myelopoiesis. Modelling of the frequency of the expression of mRNAs observed during 25 h within self-renewing cells best fitted a random model for transcriptional bursting, with events varying in duration [38]. Gata1 and Gata2 are master regulators of erythropoiesis and were ranked high as predictors of erythroid commitment in the study. The EpoR promotor contains functional Gata-binding sequences and Gata1, Gata2, or one of the other four Gata family members may be responsible for EpoR expression within erythroid-affiliated HSCs. Gata1-deficient cells, in chimeric mice and embryonic stem cell cultures, matured to the pro-erythroblast stage, and because these cells express EpoR it is unlikely that Gata1 is driving EpoR expression. Instead, studies of the myeloid FDCW2 cells (a subline of FDCP1-WEH12), in which Gata1 was expressed together with the EpoR, support the view that EpoR-mediated events contribute to Gata1 induction of a program of expression of erythroid genes [39]. Gata2 regulates the expression of the EpoR, including binding to the gene promotor, at least in pre-B acute lymphoblastic cells. The up-regulation is complex, via epigenetic, transcriptional, and post-transcriptional events, and cell context-dependent [40]. Gata2 has a pivotal role in the development of HSCs/HPCs, because a transcriptional network involving Gata2, FLII, and SCL, which recruits to the *RUNX1* enhancer, is important for the emergence of HSCs [41].

The underlying structure of chromatin is important for the translational noise within the levels of mRNAs, because this correlates with the three-dimensional organization of nuclear domains [42]. The *SATB Homeobox 1* (SATB1) *gene* encodes a matrix protein that binds nuclear matrix and scaffold-associating DNAs and SATB1 regulates chromatin structure and gene expression by means of recruiting chromatin-modelling factors. Variable levels of SATB1 correlate with the heterogeneity of HSCs regarding their distinct lineage fates [43]. There is also dynamic variation in the DNA methylation patterns across DNA, and/or activating and repressive histones modifications, that engrave the status of chromatin [44], adding to the generation of random noise within the expression of mRNAs.

Early studies examined lineage priming within single HSCs and concluded that the expression of the genes that encode the receptors for the hematopoietic cytokines is fundamental to preparing for exclusive lineage-affiliation. Investigators made use of the sensitivity of RT-PCR to measure low levels of mRNAs for genes that are lineage-affiliated within FDCP-mix cells, derived from mouse bone marrow and that self-renew and undergo multi-myeloid differentiation, and CD34^+^ mouse bone marrow cells lacking lineage surface markers. Both of these cell populations expressed cytokine receptor mRNAs at low levels. All of the FDCP-mix cells expressed the receptor for IL-3, in keeping with their IL-3-dependence, whereas a variable percentage of cells expressed the receptors for Epo (10%), M-CSF (22%), G-CSF (54%), and GM-CSF (48%). FDCP-mix cells also appeared to co-express receptors, leading investigators to the view that individual cells transit through the low-level priming of different lineage-affiliated loci [45].

## 6. Some Hematopoietic Cytokines Instruct Lineage Fate

The existence of HSCs that selectively express receptors for lineage-affiliated cytokines at a low level is highly germane to how HSCs “choose” a lineage fate. For many years M-CSF, G-CSF, GM-CSF, Epo, and Flt3L were viewed as survival and proliferation factors for lineage committed HPCs, but they are now also known to instruct cell lineage choice.

M-CSF instructs myeloid lineage fate within HSCs. Highly purified HSCs were treated with M-CSF in culture and the use of single-cell gene video imaging and expression analysis revealed that M-CSF induces the master myeloid transcription factor PU.1 and that an increased number of PU.1^+^ cells have a myeloid signature [46]. M-CSF and GM-CSF instruct macrophage and granulocyte fates, respectively, within mouse granulocye-macrophage colony forming cells (GM-CFC), as shown by culturing each of the daughter cells of GM-CFC in each factor and examining the progeny [47]. Bio-imaging approaches were used to follow the development of GM-CFC and their progeny, when cultured in the presence of either M-CSF or G-SCF, and revealed that these cytokines instruct macrophage and granulocyte fate, respectively [48].

Epo instructs erythroid fate within mouse HSCs/multipotent HPCs. Investigators used a CMV-based Epo expression vector to increase the systemic level of Epo in mice to that observed in anaemic patients. Erythroid commitment-associated genes were upregulated and megakaryocyte commitment- and granulocyte/macrophage commitment-associated genes were down-regulation within HSCs/multipotent HPCs that were purified from the mice as LSK Flt^−^. This change in the pattern of gene regulation was also observed post-treatment in LSK CD150^+^ Flt3^−^ cells with Epo in vitro. Transplantation of Epo-exposed HSCs/multipotent HPCs into sub-lethally irradiated mice generated higher numbers of erythrocytes and fewer myeloid cells. Similarly, erythroid colonies were increased when a population of marrow cells from the mice that contained both myeloid and erythroid progenitors (LSK cells) was plated in conditions for both myeloid and erythroid differentiation. The conclusion from these studies was that the instructive action of Epo extends from HSCs to HPCs [49]. As mentioned above, HPCs other than those that give rise to megakaryocytes and erythrocytes express Flt3. Flt3L transgenic mice developed anemia together with a reduction in platelet numbers. A very high level of Flt3L had therefore diverted murine LSK cells towards myeloid–lymphoid development and suppressed the generation of megakaryocyte and erythroid progenitors [50].

The above cytokines can regulate HSC/HPC survival, proliferation, and lineage commitment, but a high level of cytokine was required in the in vivo and ex vivo experimental studies to instruct cell lineage, as seen for the Flt3 ligand transgenic mice and the use of an Epo expression vector. Perhaps the level is important, whereby a low level is sufficient for survival, a raised level drives proliferation, and a further increase instructs cell lineage. It is interesting to speculate that the latter is more relevant for increasing the output of a cell type, rather than steady state hematopoiesis. In particular, lineage-affiliated HSCs enable an organism to meet an emergency requirement for a particular cell type(s). Following the acute depletion of platelets, investigators observed a rapid recruitment of platelet-biased (vWF+) HSCs, and perivascular niche cells sensed thrombocytopenia and activated platelet-biased HSCs to replenish circulating platelets rapidly [51]. During chronic and sustained in vivo erythroid stress, Epo levels are high and HSCs that display an erythroid progenitor profile, rather than multipotent HPCs, drive erythropoiesis to replenish erythrocytes quickly [52].

The cytokine requirements of multipotent and lineage affiliated HSCs are likely to be different, for example, EpoR^+^ erythroid-affiliated HSCs need to pursue their terminal erythroid development program. Niche compartmentalization of the provision of cytokine-mediated signaling would mean that there is a need for HSC sub-populations to locate to different niches in the marrow. How might they do so? SCF is a chemo-attractant for human CD34^+^ HPCs from cord and peripheral blood. Grafting of human Nalm-6 pre-B acute lymphoblastic leukemia cells into SCID mice created malignant niches that secreted SCF, which, in turn, led to the migration of the human HPCs into the niches, including HPCs that were pre-established in marrow [53]. That Epo, M-CSF, and GM-CSF are potent chemo-attractants has been largely ignored because we do not have information about whether this is the case for HSCs/HPCs. Epo increases the migration of mesenchymal stem cells [54], endothelial cells [55], and human neuroblastoma cells [56]. M-CSF is chemotactic for osteoclasts [57], monocytes [58], macrophages [59], and 32D myeloid progenitor cells transfected with the M-CSFR [60]. GM-CSF influences the migration of human neutrophils, peripheral blood monocytes, neutrophils derived from the HL60 promyeloid and myeloproliferative disorder cell lines [61], endothelial cells [62], and mesenchymal cells [63]. We might expect that cytokine receptor-bearing HSCs are responsive to the chemo-attractant action of the lineage-affiliated cytokines.

An intriguing speculation is that HSCs that express one of the lineage-affiliated cytokine receptors at a low level migrate along a gradient towards a particular niche that is rich source of the cytokine, as presented by stromal cells (Figure 2). Whilst this is technically demanding to prove, the importance is that the expression of the receptor is positively auto-regulated by cytokine binding, at least in the case of M-CSF and G-CSF (Figure 2). M-CSF binding to its receptor upregulates M-CSFR expression, via the provoked expression of the transcription factor PU.1, and G-CSF receptor (G-CSFR) signaling increases expression of the transcription factor C/EBPα, which upregulates expression of the G-CSFR (reviewed in [64]). The affiliated HSC is then “locked” into expressing the receptor and a cytokine dependence for further development.

Future advances in the field of bone marrow tissue engineering, with the means to control niche components, will no doubt facilitate gaining a better understanding of how HSCs develop towards an end cell type. This information should offer the prospect of generating large numbers of specific types of hematopoietic cells for therapeutic purposes, including from HSCs and HPCs from human pluripotent stem cells [65] or induced pluripotent stem cells (iPS). New artificial systems for replicating the physiology of marrow niches to expand HSCs and HPCs include a bone marrow-on-a-chip platform [66] and a human three-dimensional perfusion-based bioreactor system [67].

## 7. Hematopoietic Malignancies

An understanding of how certain bone marrow niches control HSC development is clearly germane to resolving what occurs when the bone marrow is given over to producing an abundance of leukemia cells. Moreover, the stem cell theory of cancer, proposed from early studies of acute myeloid leukemia, states that most leukemias arise from HSCs [68]. The leukemia stem cells (LSCs) sustain disease by generating a hierarchy of cells that often are only partially differentiated. The identification of cancer stem cells for breast, brain, lung, and gastrointestinal cancers extended the hypothesis to most, if not all, cancers arising from a tissue-specific stem cell (reviewed in [69]).

Unlike the diverse progeny of normal multipotent stem cells, the bulk cells of the hundreds of distinct cancer types resemble just one mature or partially mature cell type, even when the origin is a multipotent stem cell. This is clearly seen for chronic myeloid leukemia (CML) and acute erythroid leukemia, which arise in an HSC with the progeny belonging to just the neutrophil and erythroid lineages, respectively. Some leukemias were thought to arise in a lineage-committed HPC, but further studies revealed they actually have an HSC origin. Infant B acute lymphoblastic leukemia cells are immature B-lymphoblasts, but genome-wide analysis identified a fetal liver HSC as the origin [70]. Common acute lymphoblastic cells are B-lymphoid cells, but to replicate the human disease in mice needs HSC-like cells lacking B-cell markers [71]. In acute promyelocytic leukemia, the hallmark oncoprotein PML-RARα is present in patient HSCs, indicating an HSC origin, as reviewed in [72].

**Figure 3:**
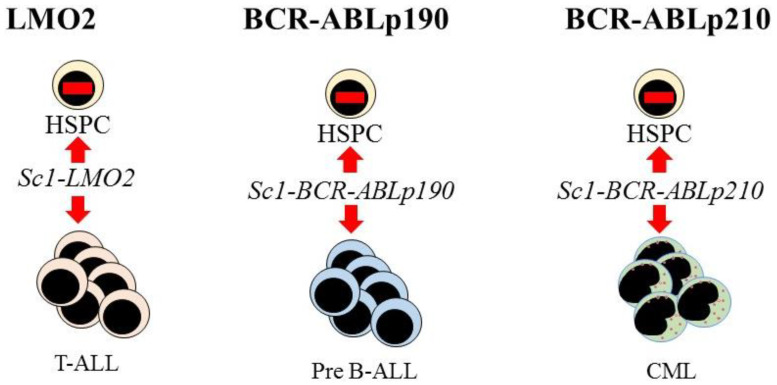
Expression of each oncogene in mouse hematopoietic stem cells leads to T-cell, B-cell, and myeloid leukemia, respectively. Targeted expression of *LMO2*, *BCR-ABLp190*, and *BCR-ABLp210* to hematopoietic stem and progenitor cells (HSPC) in transgenic mice was via placing these oncogenes under the control of the stem-cell-specific *Sca1* gene promoter. This led to murine lineage-restricted T-cell acute lymphoblastic (T-ALL), pre-B acute lymphoblastic (Pre-B ALL), and chronic myeloid leukemias (CML), respectively, with each disease typifying the human leukemia.

Further evidence supporting the view that LSCs are lineage restricted is that some oncogenes impose a lineage fate on HSCs. Investigators made use of transgenic mouse models to investigate the initiation of leukemia in HSCs. The LMO2, BCR-ABLp190, and BCR-ABLp210 oncogenes play key roles in the onset of human acute T-lymphoblastic, pre-B acute lymphoblastic, and CML, respectively. Restriction of the expression of each oncogene to HSCs, via the Sca-1 promotor, allowed investigators to determine whether these oncogenes alter the behavior of HSCs. In the mice, each oncogene led to the respective human-like lineage-restricted leukemia (Figure 3) [73,74,75]. The investigators concluded that each of the above oncogenes shapes the dynamic of lineage fate within the LSCs to constrain lineage choice to just one, which persists throughout LSC development or that manifests at a particular stage, thus restricting the bulk leukemia cells to one pathway. In either case, LSCs and their progeny lack the versatility of normal HSCs (reviewed in [76]).

The behavior of leukemia cells, and cancer cells in general, is anarchic. They have escaped from the needs of the organism as a whole because they seem to be unable to follow a pathway that is different from that which is oncogene-wired. Even so, the survival of leukemia progenitor cells remains cytokine- and stromal cell-dependent. The survival of pre-B ALL cells ex vivo is supported by IL-3 and IL-7, and stromal cells [77]. CML progenitors live in a cytokine-enriched environment, and ex vivo blocking of the influence of cytokines, by cytokine withdrawal, in addition to inhibition of bcr-Abl kinase activity, led to apoptosis [78]. The precise role(s) of niches in sustaining a substantial output of lineage-restricted leukemia cells remains to be seen.

## 8. Concluding Remarks

Stochastic modelling of lineage affiliation is highly suited to describing complex systems and HSCs as independent decision-makers, for self-renewal and cell lineage choice [79]. The influence of niches is also a critical component of the causal network in HSC decision-making. A radical revision to our view of hematopoiesis is that HSCs “sit” along a continuous spectrum of all lineage fate options. Noise within the three-dimensional organization of nuclear domains and at the epigenetic level, including the heterogeneity of chromatin accessibility and histone marks, seems to drive a low-level expression of the receptors for cytokines that are lineage-instructive. Single-lineage restricted HSCs can therefore emerge directly. Even so, hematopoiesis is flexible, because HSCs and HPCs can still take a route to a different fate, to adapt to a changing demand. The nature of the niches that lineage-affiliated and multipotent HSCs migrate to is important. Instructive cytokines may provide a chemotactic gradient to attract cells to a particular niche, where a supply of cytokine(s) enforces a pathway of development because the expression of some of the receptors is positively auto-regulated by the cytokine.

Fifty years ago, investigators observed, by electron microscopy, specialized niches for erythropoiesis, termed erythroblastic islands, that ensure autonomous and terminal-erythroid differentiation [80]. However, and as yet, our understanding of the different niches, environment signals, and intracellular signaling pathways that orchestrate how HSCs give rise to more than 100 billion blood and immune cells every day is still somewhat incomplete. The complexities of engineering ex vivo niches for hematopoiesis are the heterogeneous nature of HSCs and their supporting niche cells, together with information about the precise outcome of the cross-talk. Studies in the near future should provide the means to engineer ex vivo niches than can better replicate aspects of HSC behavior. Goals are to allow a substantial expansion of HSCs and, in particular, to resolve how HSCs “choose” a lineage fate. Findings from studies of the development of the blood and immune cells have founded our understanding of tissue-specific stems cells. A clear account of the developmental behavior of HSCs will impact on the application of stem cells *per se* in regenerative medicine.

## Data Availability

Not applicable.
